# Human mesenchymal stromal cells exert HGF dependent cytoprotective effects in a human relevant pre-clinical model of COPD

**DOI:** 10.1038/srep38207

**Published:** 2016-12-06

**Authors:** Helen Kennelly, Bernard P. Mahon, Karen English

**Affiliations:** 1 Cellular Immunology Laboratory, Institute of Immunology, Department of Biology, Maynooth University, National University of Ireland Maynooth Co, Kildare, Ireland

## Abstract

Bone-marrow derived mesenchymal stromal cells (MSCs) have potent immunomodulatory and tissue reparative properties, which may be beneficial in the treatment of inflammatory diseases such as COPD. This study examined the mechanisms by which human MSCs protect against elastase induced emphysema. Using a novel human relevant pre-clinical model of emphysema the efficacy of human MSC therapy and optimal cell dose were investigated. Protective effects were examined in the lung through histological examination. Further *in vivo* experiments examined the reparative abilities of MSCs after tissue damage was established and the role played by soluble factors secreted by MSCs. The mechanism of MSC action was determined in using shRNA gene knockdown. Human MSC therapy and MSC conditioned media exerted significant cytoprotective effects when administered early at the onset of the disease. These protective effects were due to significant anti-inflammatory, anti-fibrotic and anti-apoptotic mechanisms, mediated in part through MSC production of hepatocyte growth factor (HGF). When MSC administration was delayed, significant protection of the lung architecture was observed but this was less extensive. MSC cell therapy was more effective than MSC conditioned medium in this emphysema model.

Chronic obstructive pulmonary disease (COPD) is a progressive inflammatory disease linked to smoking and other respiratory insults, for which there is no cure. The prevalence of COPD is increasing and predicted to become the third most common cause of death worldwide by 2020^1^. COPD is characterised by airflow obstruction linked to fibrosis of the airways and destruction of the alveolar architecture resulting in emphysema^2^. These changes follow a sustained inflammatory response with an increased immune cell infiltration and elevated levels of pro-inflammatory cytokines^3^,^4^,^5^. Currently the lack of effective treatments for COPD creates a requirement for new therapies. In developing a successful therapy for COPD, a multifactorial approach is needed, to address the on-going inflammation and disease process, while promoting repair of injured tissue. In this respect human MSCs are an attractive cellular therapy, possessing potent immunomodulatory^6^,^7^,^8^ and reparative effects^9^,^10^,^11^.

To date the most successful *in vivo* application of MSCs has been for acute inflammatory events including sepsis^12^, acute renal failure^13^, myocardial infarction^14^ and acute lung injury (ALI)^15^. However, given the wide-ranging therapeutic potential of MSC, it is likely that they will be suitable for treatment of chronic diseases. An array of clinical trials have explored the therapeutic effects of MSCs in a range of diseases including, myocardial infarction, graft versus host disease and pulmonary disease^16^. On-going clinical trials are investigating the safety and feasibility and in some cases efficacy of MSCs in COPD, idiopathic pulmonary fibrosis (IPF), silicosis, bronchopulmonary dysplasia and acute respiratory distress syndrome^17^. Our work has previously demonstrated that MSCs protect against ovalbumin induced lung inflammation *in vivo*^18^. With proven benefits in lung disease models, this study sought to determine if MSCs could produce beneficial effects in a chronic disease model through promotion of repair as well as their established anti-inflammatory activities.

The trophic mediated effects of MSCs have become the focus of current therapeutic research. MSCs are capable of producing a wide range of soluble mediators that may contribute to their regenerative and protective abilities^19^,^20^,^21^; identifying these will be important in effectively utilising MSCs for clinical applications. This study, sought to investigate both a protective and reparative role for human MSCs and identify the mechanism of action involved.

Conventional murine models for human cell therapies, face confounding effects of xenoreactivity. A human relevant pre-clinical model of emphysema was used to test the efficacy of human MSCs (hMSCs) as a treatment for COPD. The pancreatic porcine elastase (PPE) emphysema model was adapted for NOD/SCID/Il-2 receptor γ-chain null mice that lack an adaptive immune system, facilitating the successful administration of hMSCs. Utilising these immunodeficient mice ensured there was no immune response initiated to the human MSCs and consequently no potential reduction in their efficacy *in vivo*. In developing the clinical relevance of the model presented in this work, an MSC dose response was carried out and the timing of administration of MSC was examined at later timepoints with delivery after establishment of tissue damage. The use of human MSCs and adaptation of the emphysema model represent more accurately the clinical course of the disease and provide a model to assess a likely future therapy. Using specific knockdown approaches, the beneficial anti-inflammatory, reparative and cytoprotective effects of MSCs administered at different disease stages were characterised and a specific soluble effector identified.

## Materials and Methods

### Cell Culture

Human bone marrow MSCs were isolated as previously described^22^ and kindly provided by collaborators in REMEDI, NUI Galway. MSC and fibroblasts were grown and maintained in complete DMEM (cDMEM, Sigma-Aldrich, Arklow, Ireland) supplemented with 10% (v/v) FBS (BioSera, Sussex, UK) and 1% (v/v) Penicillin/Streptomycin (Sigma-Aldrich) and cultured at 37 °C in 5% CO_2._ Fibroblasts were grown and maintained in cDMEM, supplemented with 10% FBS, 1% penicillin/streptomycin and 1% L-glutamine in a T75 flask at 37 °C in an incubator containing 5% CO_2._ Conditioned Medium (CM) was generated by culturing MSC or fibroblasts (1 × 10^5^/well, 6 well plate) in cDMEM for 24 h. Cells were washed with PBS and DMEM alone added for 24 h. Supernatant was collected and concentrated using Amicon Ultra Centrifugal Filters (Sigma-Aldrich).

### shRNA HGF Knockdown

HGF short hairpin RNA (shRNA) or non-silencing control plasmids were expressed in the lentiviral vector pGIPZ (Open Biosystems-Thermo, GE-Healthcare, Hatfield, UK). HGF plasmids were transfected into HEK293T cells with the packaging plasmid Trans-Lentiviral packaging mix using Mirus-TransIT-293T transfection reagent. HEK293t cells were cultured in high glucose DMEM (Sigma Aldrich) supplemented with 10% (v/v) FBS, 1% (v/v) penicillin/streptomycin (Invitrogen). After 24 h transfection, 30% FBS DMEM was added. 48 h after transfection, the supernatant containing the HGF shRNA lentivirus was collected. Human MSCs (passage 2) were transduced with the viral medium for 72 h followed by positive selection using 4 μg/ml Puromycin (Sigma-Aldrich).

### Elastase induced mouse model of COPD

NOD.Cg-Prkdc^scid^IL2^tmlWjl^/Szj mice (NOD-SCID IL-2rg^null^) (NSG) (Jackson Laboratories, Bar Harbour, ME,) were utilised for the elastase model. The treatment groups for the *in vivo* study included PBS only control groups, elastase only groups and elastase + MSC treatment groups. Mice received 30 μg of porcine pancreatic elastase (Sigma-Aldrich) in 50 μl PBS (or PBS only for control groups) by intranasal instillation per dose (total -6 doses over 2 weeks). MSCs (or PBS for control) were administered i.v. (intravenously) on day 0 (early administration), day 7 (mid-treatment administration) or day 14 (delayed administration). Initially a dose response was carried out using MSC at 0.1, 5, 25 or 125 × 10^3^ cells/g. For all of the other experiments MSCs were administered i.v. at a dose of 25 × 10^3^ cells/g mouse weight such that a 20 g mouse would receive 5 × 10^5^ cells/mouse.

### Histopathology

Lungs were harvested, formalin fixed and paraffin embedded. Tissue was sectioned and stained with either Mason’s Trichrome or Weigert’s Iron Hematoxylin and Eosin (Sigma-Aldrich) stain. Airway pathology was examined under a light microscope with at least three fields of view taken for 3 sections per mouse, with 5 mice per group. A blinded semi-quantitative scoring system utilising the Ashcroft scale was used to assess collagen deposition/fibrotic burden in the lung^23^. Mean linear intercept (MLI) and alveoli number were measured as previously described^24^ using image J software. The distance between the alveolar walls was measured by drawing a line across each alveoli from wall to wall and recording the length for each measurement. This was carried out for three fields of view with three sections from each mouse lung. Alveoli number was determined by the number of measurements made for the MLI.

### TUNEL assay

TUNEL assay was carried out using the *In Situ* Cell Death Detection Kit according to the manufacturers instructions (Roche, Dublin, Ireland). 5 μm tissue sections were fixed onto charged glass slides (VWR, Dublin, Ireland) at 50 °C overnight. Wax was removed and tissue rehydrated. Sodium Citrate antigen retrieval was performed. Sections were incubated at 37 °C for 1 h in humid conditions with 20 μl label and enzyme solution. Tissues were counterstained with DAPI (300 nM) (Sigma-Aldrich).

### Real-time PCR analysis

TriReagent (Life Technologies, Cheshire, UK) was used to isolate total RNA and samples were reverse transcribed to cDNA using Tetro cDNA synthesis kit (Bio- Line, London, UK). Real-time PCR analysis was performed using SYBR Green reagent (Sigma-Aldrich) and specific primers were utilized to determine gene expression. The following PCR predesigned primers were used, IL-1β F-GGATGATGATGATGATAACCTGC, R-CATGGAGAATATCACTTGTTGG, IL-6 F-ACAGTTGCCATGTAGACC, R-TTCTGTGACTCCAGCTTATC, TNFα F-GGATGAGAAGTTCCCAAATC R-TGAGAAGATGATCTGAGTGTG and GAPDH F-AGGGATTTGAATCACGTTTG, R- TTTACTGGCAACATCAACAG used as the house keeping gene.

### Ethical approval

All procedures involving animals were carried out by licensed personnel. Ethical approval for all work was received from the research ethics committee of Maynooth University. All procedures were carried out under an approved Department of Health animal licence.

### Statistics

Statistical analysis was performed using GraphPad Prism™ software (GraphPad, San Diego, CA). The student’s paired t-test was used when statistical analysis was required between two experimental groups. One way ANOVA was used for multiple experimental group comparison. Data are presented as the ± standard error of the mean (SEM). P-values of p < 0.05 (*), p < 0.01 (**) or p < 0.001 (***) were considered statistically significant.

## Results

### Protection by early administration of hMSC against elastase induced emphysema in a human relevant mouse model is dose dependent

**Figure 1:**
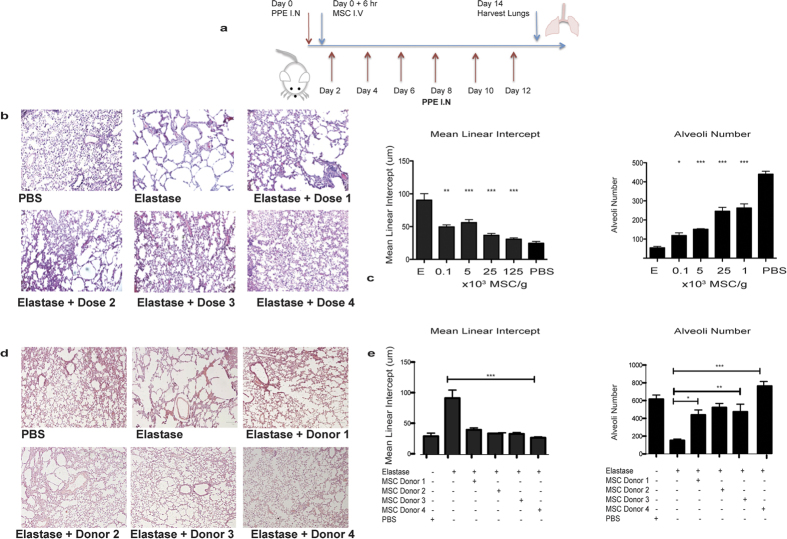
Early administration of hMSCs protects against elastase induced emphysema in a dose dependent manner. (**a**) Elastase (30 ug) was delivered intranasally (I.N) to mice to induce emphysema like changes in the lung. hMSCs were administered (once) intravenously 6 hours after the first elastase instillation. Further doses of elastase were given every 2 days over the course of two weeks, until the lungs were harvested for analysis on day 14. (**b**) Transverse tissue sections were cut and H&E stained and images were taken using phase contrast microscopy at a magnification of 10X. Representative images show the tissue and alveoli structure in control PBS lungs, elastase lungs, hMSC dose 1 (0.1 × 10^3^ cells/g), hMSC dose 2 (5 × 10^3^ cells/g), hMSC dose 3 (25 × 10^3^ cells/g) and hMSC dose 4 (125 × 10^3^ cells/g) treated lungs. (**c**) The mean linear intercept and alveoli number was calculated from the H&E stained lung sections for each group, with three images from each lung used to calculate an average using Image J software. Data presented shows the mean linear intercept and alveoli number for all doses. MSCs isolated from different donors were administered (once) at the optimal dose (25 × 10^3^ cells/g) intravenously 6 hours after elastase on day 0. (**d**) Representative images show alveoli structure from PBS lungs, elastase lungs, MSC donor 1 lungs, MSC donor 2 lungs, MSC donor 3 lungs and MSC donor 4 treated lungs. (**e**) Data presented shows mean linear intercept and alveoli number. N = 5 mice for all groups. Data expressed as means ± S.E.M. Statistical analysis was carried out using one way ANOVA test. *p < 0.05, **p < 0.01, ***p < 0.001.

*In vitro* and conventional animal models are limited substitutes for examining human emphysema. A pre-clinical model of COPD was used to elucidate the effect of human MSC therapy on COPD *in vivo.* Immunocompromised NSG mice were used to facilitate the study of hMSCs as potential cell therapy candidates. Emphysema like changes were induced in the lung through elastase instillation delivered intranasally over two weeks (Fig. 1a).

In a preventative disease model, MSCs were administered at increasing doses to determine an optimal cell dose. Lung damage was quantified through measurement of the mean linear intercept and alveoli number. In comparison to healthy (PBS control) lungs, elastase caused significant damage to alveoli structures; with a breakdown in alveolar walls and decrease in alveoli number. Following hMSC administration at day 0, a protective effect was observed (Fig. 1) with reduced alveolar damage and reduced alveoli number loss.

Importantly, an hMSC dose as low as 0.1 × 10^3^/g (equivalent to 1 × 10^5^/kg) significantly decreased the mean linear intercept and increased the alveoli number in comparison to untreated elastase exposed mice (Fig. 1b,c). While all hMSC doses significantly improved mean linear intercept and alveoli number, the strongest effects were observed with 25 × 10^3^ cells/g and with 125 × 10^3^ cells/g with no significant difference detected between these groups (Fig. 1b,c). The lower cell dose of 25 × 10^3^ cells/g was used for all further *in vivo* studies.

The emphysema model was used to determine if MSC protective effects were donor specific. Elastase induced damage was prevented by four different hMSC donors (Fig. 1d). All MSC donors significantly reduced the mean linear intercept and increased alveoli number (Fig. 1e).

### hMSC reduce fibrosis, apoptosis and inflammation in a humanised mouse model of emphysema

**Figure 2:**
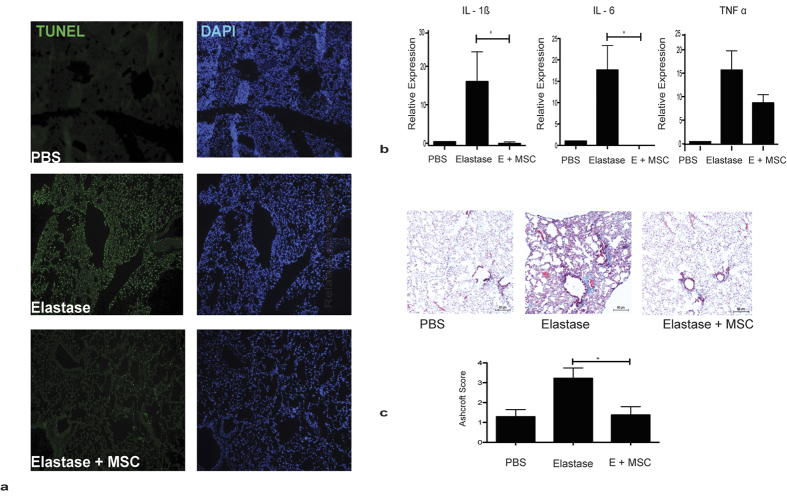
hMSCs reduce fibrosis, apoptosis and inflammation in a mouse model of emphysema. (**a**) Transverse lung sections from PBS control, elastase or hMSC treated elastase mice were stained for DNA strand breaks by TUNEL assay, specifically with fluorescein dUTP. Lung sections were counterstained with DAPI and representative images are shown at 10X magnification. (**b**) The mRNA levels of IL-1β, IL-6 and TNFα were measured by qRT-PCR, changes in expression were measured relative to the house keeping gene GAPDH. (**c**) Representative images of tri-chrome stained transverse lung sections are shown for PBS, Elastase and Elastase + MSC (E + MSC). Histological scoring was carried out on tissue sections using the Ashcroft scale with three images from each lung used to calculate an average. N = 4 mice for all groups. Data expressed as means ± S.E.M. Statistical analysis was carried out using one way ANOVA analysis test *p < 0.05.

Increased levels of alveolar epithelial apoptosis are a significant feature of emphysema^25^,^26^. Human MSC secrete a range of cytoprotective factors, with roles in protection and repair^11^,^19^,^20^. We have shown that soluble factors secreted into media (conditioned medium (CM)) by MSC (hMSC CM) can protect against alveolar apoptosis *in vitro* (Supp. Fig. 1). To determine if hMSC administration protected airway epithelium from apoptosis *in vivo*, a TUNEL assay was performed on tissue derived from the elastase induced emphysema model described above. As expected, elastase administration over the two weeks induced high levels of apoptosis when compared to control PBS groups (Fig. 2). hMSC treatment markedly reduced apoptosis in the lungs (Fig. 2a). To examine how hMSCs were exerting their protective effects in the lung, key inflammatory cytokines (IL-1β, IL-6 and TNFα) associated with COPD were examined by qRT-PCR. In elastase exposed lungs treated with hMSCs, there was a significant reduction in IL-1β and IL-6 compared to elastase only lungs (Fig. 2b) suggesting that hMSCs affect production of these cytokines.

The fibrotic response of lung tissue remodelling in COPD patients is also a significant contributor to the pathological changes associated with COPD^27^. To determine if hMSCs could reduce fibrosis, collagen deposition was assessed in the elastase model through Tri-chrome staining of lung sections using the Ashcroft scale. Alveolar wall thickening and collagen deposition was observed in elastase exposed airways (Fig. 2c). In contrast hMSC treatment of mice exposed to elastase resulted in reduced collagen deposition in the lungs (Fig. 2c) indicating a potential anti-fibrotic role for hMSCs when delivered intravenously on day 0.

### hMSC administration during or after establishment of emphysema maintain a level of therapeutic efficacy

**Figure 3:**
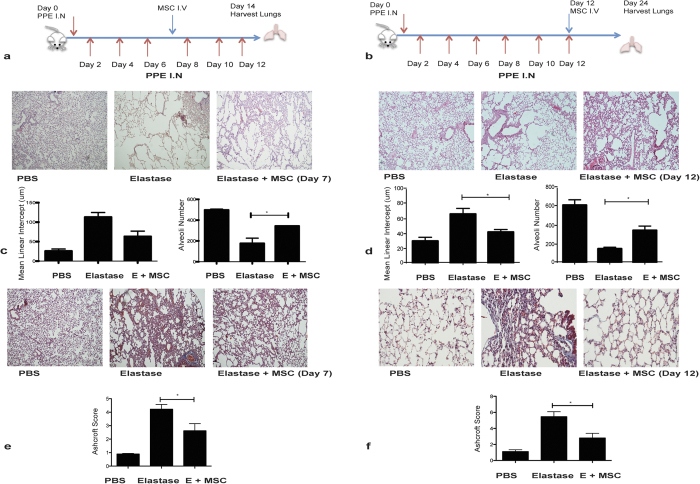
hMSC administration during or after establishment of emphysema maintain a level of therapeutic efficacy. Timeline of mid-treatment (**a**) or delayed (**b**) model, with MSC administered on day 7 (**a**) or day 12 (**b**). Tissue sections were cut and H&E stained, and images taken at 10X magnification. Representative images show alveoli number and structure from PBS, Elastase and Elastase + hMSC- mid (Day 7) or delayed (Day 12) treatment lungs. Mean linear intercept and alveoli number were calculated from H&E stained lung sections using Image J software for (**c**) mid-treatment or (**d**) delayed treatment. Tissue was tri–chrome stained and images were taken at 10X magnification to assess fibrotic burden. Representative images show PBS, Elastase and Elastase + MSC (Day 7 or Day 12) lungs. Histological scoring was carried out on tissue sections using the Ashcroft scale with three images scored for each lung (**e**) mid-treatment and (**f**) delayed treatment. N = 4 mice for all groups and presented as mean ± S.E.M. Statistical analysis was carried out using one way ANOVA analysis test *p < 0.05.

The data above show that hMSCs have *in vivo* therapeutic benefit early post elastase exposure, however in COPD a potential therapy will have to be effective post induction of damage. To more closely mimic the clinical course of the disease and potential therapy in this model, administration of hMSCs was delayed to the mid-way point of the study at day 7 (Fig. 3a) ensuring that there were emphysematous changes induced in the lung before MSC administration.

Day 7 hMSC treatment resulted in improved lung pathology. There were marked differences in the number of alveoli observed and in tissue structure. The mean linear intercept was significantly decreased; with a significant increase in alveoli number after hMSC treatment (Fig. 3b). Notably, hMSC therapy caused a reduction in collagen deposition (Fig. 3c). Importantly these findings demonstrate that hMSC were able to exert cytoprotective effects and promote repair of previously injured tissue at a more advanced stage of the disease.

To test this effect further, a treatment model was designed to reflect the clinical course of established COPD and delayed therapy. This required delivering the entire course of elastase administration (6 doses of PPE) over two weeks without any hMSC treatment ensuring full development of emphysema (and fibrosis) in the lungs before hMSC delivery. hMSCs were delivered on day 12 and lungs were harvested for analysis on day 28 (Fig. 3d).

Tissue damage was assessed by H&E staining. On day 28, elastase exposed lungs displayed tissue damage and alveolar destruction. While hMSC mid-treatment facilitated repair in the lung (Fig. 3c), this improvement was not as extensive as the protective effect observed previously (Fig. 1). Delayed hMSC therapy significantly reduced the mean linear intercept and preserved the alveoli number in comparison to elastase exposed lungs (Fig. 3d). Substantial collagen deposition had developed in the elastase exposed lungs by day 28, however hMSC therapy administered at delayed timepoints, significantly reduced collagen deposition (Fig. 3e,f). These data suggest that human cell therapy might be possible in COPD despite existing lung damage.

**Figure 4:**
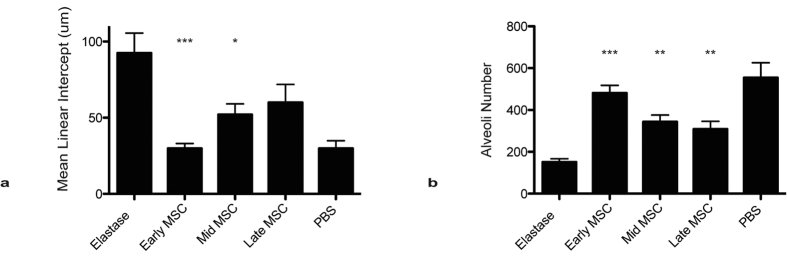
Early hMSC treatment is more efficacious that delayed MSC treatment. Data from the *in vivo* studies (Figs 1 and 3) were analysed and mean linear intercept and alveoli number measured using Image J software. N = 4 mice for all groups and presented as mean ± S.E.M.

A compaison of the effectivness of hMSC delivery at the different timepoints was made (Fig. 4). Direct compaison between the quantitative measurements of both the mean linear intercept (Fig. 4a) and alveoli number (Fig. 4b) showed the reduced effictiveness of hMSC treatment when delivered later in the course of the disease. However improvement in comparison to elastase only groups was still achieved with the later MSC administrations.

### Soluble hMSC derived factors can have a protective effect in a humanised mouse model of emphysema

**Figure 5:**
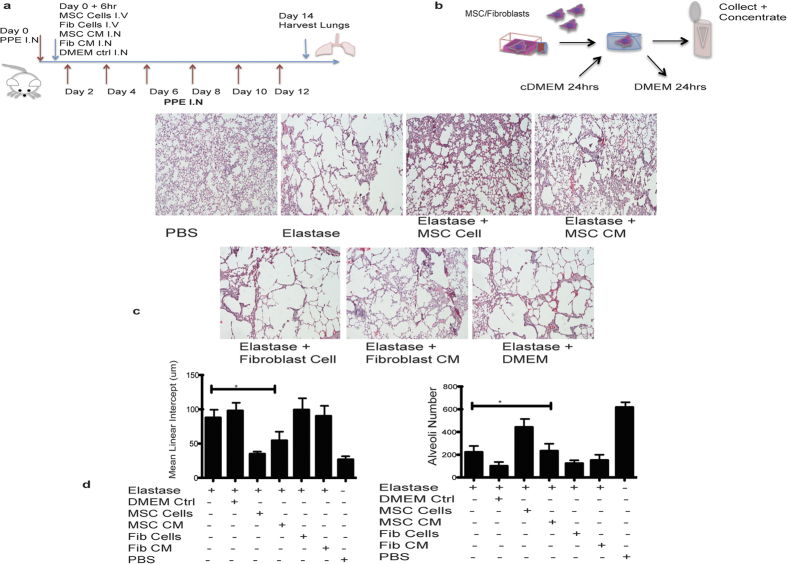
Soluble hMSC derived factors can have a protective effect in a humanised mouse model of emphysema. (**a**) Timeline (**b**) Generation of conditioned medium. Control DMEM, human fibroblast or MSC Conditioned media (CM) was administered intranasally (IN) on day 0 and compared to intravenous (IV) administration of MSC cells or fibroblast cells (25 × 10^3^/g) on day 0 in elastase treated mice, timeline shown. (**c**) Transverse tissue sections were cut and H&E stained. Images were acquired using phase contrast microscopy at 10X magnification. Representative images show the airspace and alveolar structure in PBS, Elastase, Elastase + hMSC Cells, Elastase + hMSC CM, Elastase + DMEM, Elastase + Fibroblast Cells and Elastase + Fibroblast CM treated lungs. (**d**) Mean linear intercept and alveoli number were calculated from H&E stained lung sections using Image J software with three sections from each lung. N = 4 mice for all groups. Data expressed as means ± S.E.M. Statistical analysis was carried out using one way ANOVA analysis test *p < 0.05.

Utilising hMSC conditioned medium (CM), Supp. Fig. 1 demonstrates the *in vitro* cytoprotective effects of hMSC derived soluble factors. To examine the effect of hMSC derived trophic factors *in vivo*, the therapeutic efficacy of hMSC cells was compared to that of concentrated hMSC CM in the elastase model (Fig. 5a). It was important to demonstrate that any protective effects associated with CM were specific to CM derived from hMSC and similarly that protection associated with delivery of cells was specific to hMSC. In order to examine this, DMEM medium and CM from human fibroblasts was used for comparison. In addition, to demonstrate the specificity of the protective effects mediated by hMSC, a human fibroblast control cell population was also utilised. hMSC or human fibroblast CM was generated in DMEM alone for 24 h (Fig. 5b). Control groups receiving; fibroblast CM, fibroblast cells, or DMEM alone were also compared to mice receiving hMSCs intravenously. H&E staining of lungs demonstrated a cytoprotective effect for hMSC cell therapy as expected, while MSC CM also displayed some cytoprotection, but this protective effect was not as extensive as cell therapy. This difference could be due to persistence of trophic factors in the conditioned medium or might indicate that hMSC are acting via trophic and non-trophic mechanisms to exert their cytoprotective effects. Control groups (DMEM alone, fibroblast CM or fibroblast cells) demonstrated no cytoprotective effect (Fig. 5c,d).

### HGF is required for hMSC therapeutic efficacy in emphysema

**Figure 6:**
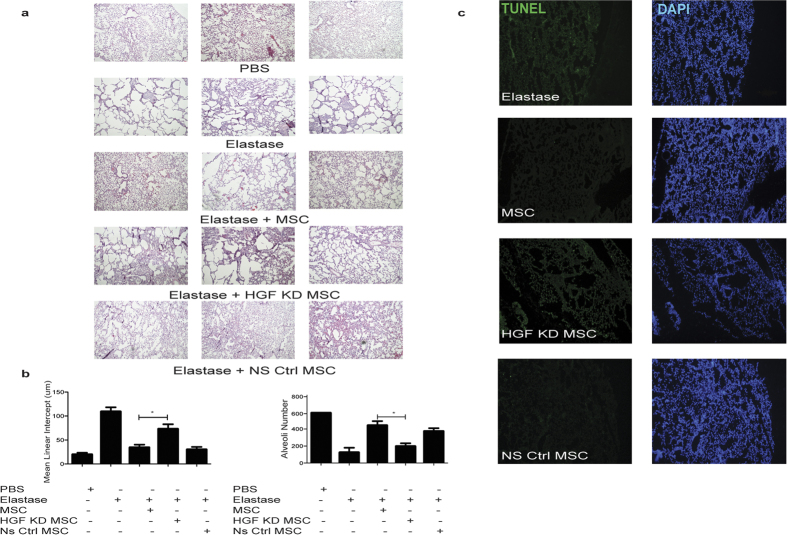
HGF is required for hMSC therapeutic efficacy in emphysema. Human HGF knockdown (KD) hMSCs, non-silencing control (NS) hMSCs or unmanipulated hMSCs were administered on day 0 of the elastase model. Lungs were harvested, formalin fixed and paraffin embedded. (**a**) Lung sections were H&E stained and images taken at 10X magnification. Representative images show PBS, Elastase, hMSC, HGF KD hMSC and NS control MSC groups. (**b**) The mean linear intercept and alveoli number were calculated from three H&E stained tissue sections from each mouse lung using Image J software. Data expressed as means ± S.E.M. Statistical analysis was carried out using one way ANOVA analysis test *p < 0.05. (**c**) Transverse lung sections were stained for DNA strand breaks by TUNEL assay, specifically with fluorescein dUTP. Lung sections were counterstained with DAPI. Representative images show Elastase, MSC, HGF KD MSC, and NS ctrl MSC. N = 4 mice for all groups.

The study demonstrating protective effects of MSC-CM (Fig. 5) and the *in vitro* assay (Supp. Fig. 1) suggested a potential role for MSC-derived soluble factors in protection against elastase induced emphysema. HGF was a likely candidate for such a role^28^,^29^. In order to test this hypothesis, HGF knockdown (KD) hMSCs were generated through shRNA for *in vivo* studies. Specifically this study sought to investigate the role for HGF in MSC cell mediated protection against elastase induced emphysema. HGF KD hMSC expressed the typical hMSC surface markers and had a significantly reduced capacity to produce HGF (Supp. Fig. 2). H&E staining demonstrated that the cytoprotective effect observed with hMSCs was reduced in HGF KD hMSCs. The preservation of alveolar structure observed with MSC treatment was decreased, with an increased destruction of alveoli walls observed. Non silencing (NS) control hMSCs showed similar cytoprotective abilities to hMSCs (Fig. 6a,b). The reduced cytoprotective ability in knockdown hMSCs indicates that sustained production of HGF may play an important role in cytoprotection in this model of COPD therapy.

The cytoprotective effect of hMSC might also be due to reduced apoptosis in elastase exposed lungs (Fig. 2a). As expected, hMSCs had an anti-apoptotic effect in the lungs, reducing the epithelial damage induced by elastase exposure whereas treatment with HGF KD hMSCs did not reduce the apoptosis in the lungs to the same extent as hMSCs or NS control hMSCs (Fig. 6c). Consequently, the anti-apoptotic effect of MSC derived HGF shown *in vitro* (Supp. Fig. 1) translated to the *in vivo* model, clarifying the mechanisms by which MSC support cytoprotection.

## Discussion

MSC have emerged as novel cell therapies, due to their established immunomodulatory and anti-inflammatory effects, with potential to be utilised therapeutically. Given that hMSC have been shown to mediate anti-inflammatory effects^8^, reduce oxidative stress, reduce fibrosis and enhance repair^30^ it seems logical that hMSC might represent a suitable and efficacious therapy for COPD. Key pathological mechanisms of epithelial damage and wound formation occur in COPD, eventually leading to development of emphysema^31^,^32^. This study has shown that hMSC possess potent cytoprotective and reparative abilities that may limit these activities. hMSC CM inhibited airway epithelial cell apoptosis, while also promoting repair and proliferation via paracrine signalling, supporting other studies highlighting protective effects of hMSC derived soluble mediators^33^,^34^.

Building on these beneficial properties of MSCs, an elastase induced emphysema model was used to examine MSC therapy. Of particular interest for the study of human therapies, this elastase model was performed in NSG mice, thereby creating a human relevant model of COPD, facilitating the investigation of human MSC therapy without the problems associated with xeno-recognition and rejection. The model of repeated elastase exposure was more reflective of smoking induced disease in humans and superior to single dose murine models in that disease induction was cumulative. This is supported by evidence from the literature demonstrating that repeated administration of elastase leads to enhanced alveolar destruction and the development of pulmonary arterial hypertension^30^,^35^,^36^. A macrophage dysfunction in the NSG mice is a caveat for this study given the key role played by macrophages in the remodelling process associated with emphysema. Despite this defect, this model approach is a significant advance in that it allows comparison to mouse models (as it displays many of the features typical of mouse models of elastase induced emphysema) but moves to study the genuine human effector mechanisms. The overall goal of this research was to utilize this human relevant pre-clinical model to investigate the effect of human MSC on elastase induced emphysema. Although a number of studies have demonstrated the capacity for rodent MSCs to protect in models of elastase and cigarette-smoke induced emphysema^30^,^37^,^38^,^39^,^40^,^41^, few studies have used human MSC. The first of these studies did not examine therapeutic efficacy but focused on tracking of MSC in an elastase model *in vivo*^42^. The second study demonstrated efficacy of human tubal-derived MSC in combination with low level laser therapy in CS-induced COPD^43^. A third study demonstrated relatively modest protection mediated by two doses of human MSC in a CS-induced model^44^. Therefore, this is the first study to comprehensively investigate the optimal dose, timing, efficacy of different donors and the mechanism of action involved in human MSC protection against emphysema in a human relevant pre-clinical model. Initially this work sought to establish the optimal dose for human MSC therapy in this model. While studies have compared different rodent MSC sources^30^ and delivery routes^30^,^45^ this is the first study to demonstrate a dose response. In line with hMSC dose selection based on hMSC/kg weight for human trials, a dose dependency study was performed based on animal weight. Comparison between the dosing study here and standard doses of MSC for patient treatment predicts that a lower dose might achieve efficacy based on this model. In a clinical trial for MSC treatment of COPD the cell dose was 100 × 10^6^ cells per infusion with 4 doses administered per patient^46^. For an average patient weight of 80 kg this represents a dose of 1.25 × 10^6^ MSC/kg per administration and a total of 5 × 10^6^ MSC/kg (over 4 administrations). Importantly this study demonstrated efficacy of a single administration of human MSC therapy with a dose as low as 0.1 × 10^3^/g which corresponds to 1 × 10^5^/kg. However, efficacy was significantly enhanced at higher doses and in a dose dependent manner with the optimal dose identified as 25 × 10^3^/g. A recent clinical trial has shown that a relatively low dose of hMSC was ineffective in COPD highlighting the importance of defining the optimal dose^46^. The data presented here supports the requirement for a dose escalation study in COPD patients to identify an optimal MSC dose and provides a parallel laboratory model to standardise future therapies.

Inevitably there will be some variation in donor hMSC populations due to differences in age, gender and health status^47^, with a potential significant impact on clinical outcomes. Utilising 25 × 10^3^ cells/g this study investigated the effect (and reproducibility) of hMSC donor variation on therapeutic outcome. Notably, this work demonstrated comparable cytoprotective effects for different human MSC donors.

Examining this cytoprotective effect, further demonstrated an anti-inflammatory effect of MSCs, reducing expression of IL-1β, IL-6 and TNFα in elastase lungs. This is in agreement with other studies demonstrating a reduction in IL-1β and IL-6 in emphysema models^48^ mediated by MSC. These cytokines are present at significantly increased levels in COPD patients with a further increase during an exacerbation^49^. This anti-inflammatory effect may be mediated by HGF inhibition of NFκB signalling in the lungs^50^. Furthermore, HGF has been shown to downregulate IL-6^51^ and enhance production of the natural IL-1receptor antagonist; IL-1RA^52^. Other studies have demonstrated the capacity for MSC to downregulate keratinocyte-derived chemokine (KC), TGF-β and COX-2-PGE-2 in rodent models of emphysema^30^. Although no mechanism was identified in MSC reduction of KC and TGF-β^30^, a partial role for p38 and ERK MAPK pathways was demonstrated in MSC downregulation of COX-2 in a COPD model^39^. Apoptotic cells have been observed in lung tissue and BALF samples of COPD patients^25^,^26^,^53^, and in animal models^31^,^54^. In line with other studies^30^,^41^ an anti-apoptotic effect of MSC was identified *in vitro* and encouragingly, this was also detected *in vivo.* A marked reduction in apoptotic cells in emphysematous lungs was evident after MSC treatment (day 0) and correlated with an improvement in lung pathology. Although more commonly associated with fibrotic lung diseases such as IPF, abnormal collagen remodelling and development of fibrosis is a component of COPD^27^,^55^. In contrast to the study carried out by Peron *et al*. showing a requirement for low level laser therapy in combination with human tubal derived MSC^43^, Antunes *et al*. observed a reduction in collagen fibre deposition mediated by mouse MSC^30^. Our study extends these findings demonstrating the capacity for early, mid or delayed treatment to significantly reduce collagen deposition in the elastase model when bone marrow derived hMSC are used.

To better reflect the clinical course of the disease (and therefore treatment timelines) the *in vivo* study timelines were modified, delivering MSC therapy at later stages to reflect the clinical course of COPD. MSC treatment on day 7 (mid) or day 14 (delayed) resulted in significant cytoprotective and anti-inflammatory effects, with lung alveolar architecture protected from elastase damage. An important confounding consideration in this delayed (28 day) model is the resolution of tissue injury in mice after elastase instillation has stopped. Whilst an overall reduction in elastase damage compared to the previous models was observed, there was still a significant improvement in mean linear intercept and alveoli number as well as reduction in collagen deposition achieved with late MSC therapy supporting findings in other COPD models^30^,^38^,^39^.

It is evident that hMSC have the capacity to exert potent cytoprotective effects particularly in acute incidences of inflammation and tissue damage. While they improve lung pathology at early, mid and late treatment timepoints, the degree to which hMSC achieve this varies. The benefits of early hMSC administration are likely due to the significant inflammation they encounter in injured tissue. The immunosuppressive abilities of MSC are enhanced by inflammatory cytokine stimulation^7^. Therefore it is likely that delivery of hMSC at the height of inflammation would improve efficacy as we have seen in other systems^8^.

A clinical trial of MSC for the treatment of COPD appears to confirm this enhanced effect of MSC therapy for acute inflammatory mediated diseases. Using allogeneic hMSCs, an early significant difference in levels of circulating c-reactive protein (CRP) was observed^46^, however, no differences in frequencies of exacerbations or worsening of disease were found. Thus, while hMSCs can reduce inflammation early after administration, the tissue damage can be so extensive that repair of the injured tissue is insufficient to make a difference therapeutically^26^. However, it is important to note that a dose escalation study has not yet been performed in a clinical trial for COPD and it may be that an optimal dose of MSC has not yet been identified for clinical efficacy. This model may help resolve that issue.

The *in vitro* work demonstrated the role of paracrine mediated effects by hMSC CM and thus CM itself was evaluated therapeutically *in vivo* demonstrating some efficacy for hMSC CM but not fibroblast CM or cells in comparison to non MSC CM (DMEM) treated elastase. This is in line with other studies demonstrating a protective effect mediated by MSC CM in lung injury models^19^,^20^. A protective effect for MSC-CM was also shown by Huh *et al*., however, that study administered 10 doses of MSC CM intravenously (and did not include non MSC CM controls)^37^ in comparison to the single dose administered intranasally in our study. In our study, MSC cellular therapy provided a significant improvement on the conditioned medium treatment. In contrast the Huh *et al*. study demonstrated comparable efficacy between a single dose of rat MSC and 10 doses of rat MSC CM^37^. The differences in these findings are likely associated with persistence of trophic factors or indicate that MSC may be acting through multiple (trophic and non-trophic) mechanisms *in vivo.*

There is a consensus that trophic factors such as VEGF and HGF may be responsible for many of the protective and tissue reparative effects of MSC. Utilising animal models, the beneficial effects of HGF in emphysema have previously been observed^28^,^29^. Importantly, while other studies have shown increased levels of VEGF or HGF following MSC therapy^30^,^38^,^40^,^48^, none of these studies utilised blocking or knockdown studies to verify the importance of either cytokine in hMSC cells. This is the first study to unequivocally demonstrate an important role for hMSC derived HGF in protection against lung injury *in vivo*.

MSC exerted significant anti-apoptotic effects in the elastase exposed lungs, however this effect was reduced with HGF knockdown MSC. Notably, the potent anti-apoptotic effect of MSC mediated against elastase induced emphysema has been validated both *in vitro* and *in vivo* with the importance of HGF signalling in the process established. This data suggests that apoptosis is a destructive component of the process of epithelial loss associated with emphysema and that HGF is a key anti-apoptotic factor secreted by MSC. In line with these findings, we and others have demonstrated an important role for HGF alone^56^ or in combination with PGE2^28^ in mediating anti-fibrotic effects in pre-clinical models of IPF and irradiation induced lung injury. This is in direct contrast to recent findings demonstrating a partial role for MSC downregulation of COX-2-PGE-2 in alveolar macrophages in a CS-induced emphysema model. This suggests that the trophic factors or mechanism of action utilised by MSC may be dependent on the particular disease environment as there are important differences in the pathophysiology of fibrotic disease and emphysema.

In conclusion we have developed a model that allows assessment of hMSC therapy for COPD. Using this and knockdown approaches we show that hMSCs can protect against COPD and reduce damage in part through HGF. Importantly, this study demonstrates the efficacy of hMSC therapy in a dose dependent manner and after the establishment of emphysema and supports the therapeutic potential of hMSC therapy for COPD.

## Additional Information

**How to cite this article**: Kennelly, H. *et al*. Human mesenchymal stromal cells exert HGF dependent cytoprotective effects in a human relevant pre-clinical model of COPD. *Sci. Rep.* **6**, 38207; doi: 10.1038/srep38207 (2016).

**Publisher's note:** Springer Nature remains neutral with regard to jurisdictional claims in published maps and institutional affiliations.

## Supplementary Material

Supplementary Information
