# Supplementary material for: Human mesenchymal stromal cells exert HGF dependent cytoprotective effects in a human relevant pre-clinical model of COPD
Source: Sci Rep. 2016 Dec 6;6:38207. doi: 10.1038/srep38207 (PMC5138599; doi:10.1038/srep38207)
Supplement: Supplementary Information [file srep38207-s1.pdf]

Supplementary Data

Human mesenchymal stromal cells exert HGF dependent cytoprotective effects in a human relevant pre-clinical model of COPD.

Helen Kennelly[1], Bernard P. Mahon[1] and Karen English[1]

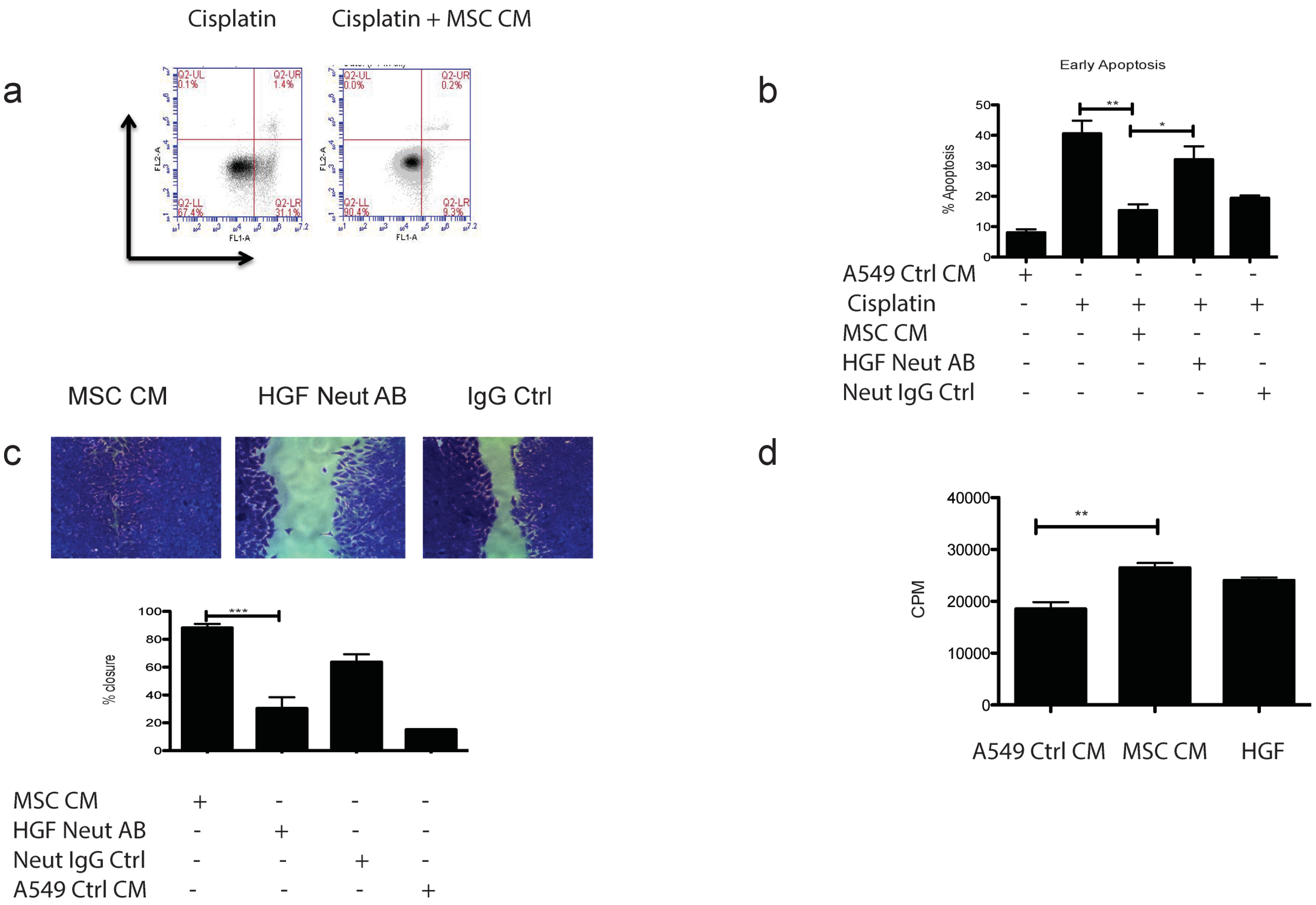
a
Cisplatin
Cisplatin + MSC CM
Q2-UL 0.1%
Q2-UR 1.4%
Q2-LL 67.4%
Q2-LR 31.1%
Q2-UL 0.0%
Q2-UR 0.2%
Q2-LL 90.4%
Q2-LR 9.3%
FL2-A
FL1-A
b
Early Apoptosis
% Apoptosis
A549 Ctrl CM
Cisplatin
MSC CM
HGF Neut AB
Neut IgG Ctrl
c
MSC CM
HGF Neut AB
IgG Ctrl
% closure
MSC CM
HGF Neut AB
Neut IgG Ctrl
A549 Ctrl CM
d
CPM
A549 Ctrl CM
MSC CM
HGF

Supplementary Figure 1.hMSC derived soluble factors protect against apoptosis and enhance wound healing *in vitro* in a HGF dependent manner. For all assays $1x10^5$ A549 cells were seeded into a 24 well plate and cultured until confluent. For the apoptosis assay: 50 µg/ml cisplatin was added to cells for 24 hours to induce apoptosis. MSC conditioned medium (CM) was generated by culturing control media with $1x10^5$ MSC overnight in a 24 well plate. HGF was inhibited through addition of a HGF neutralising antibody (5ug/ml). CM was added to appropriate wells for 24 hours. Apoptosis was determined by flow cytometric measurement of annexin V and PI (A) and percentage of early apoptotic cells measured (Annexin V^+ PI^-) (B). For the scratch assay, cells were scratched and conditioned media added. HGF was inhibited in MSC CM through addition of a neutralising antibody. After wound closure, cells were methanol fixed and stained with crystal violet and the percentage wound closure was calculated using Image J software (C). For the proliferation assay, cells were scratched and ctrl or MSC CM added. Recombinant HGF at 10ng/ml was added as a positive control. [3H] – Thymidine was added to each well and incubated for 2 days. After 2 days cells were lysed and combined with 2ml of β–scintillation counter liquid. Proliferation was measured as counts per minute (CPM). Data statistically analysed by ANOVA. p*<0.05, p**<0.01, p***<0.001. N=4 for all groups

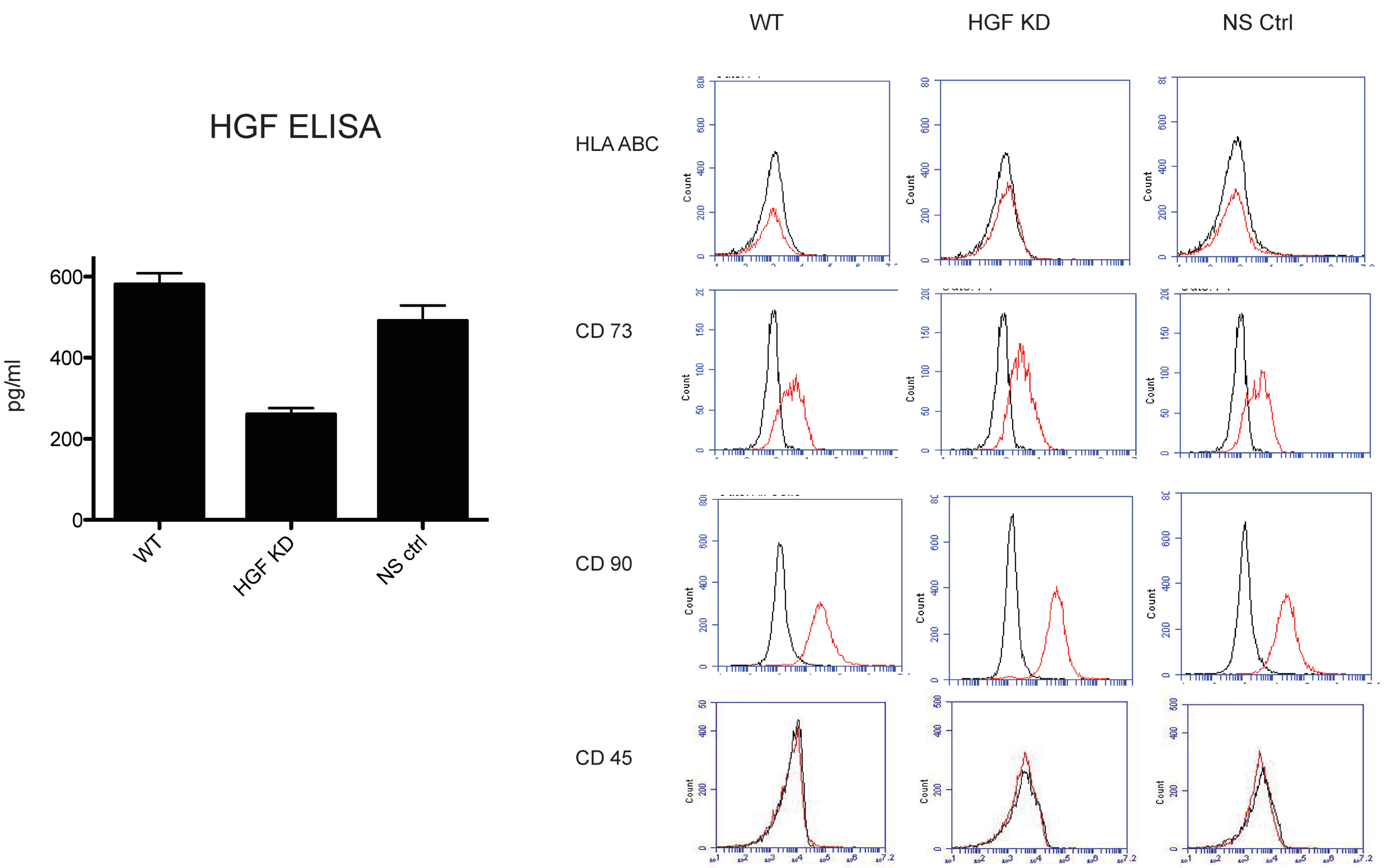

Supplementary Figure 2. HGF KD Cell Characterisation. HGF knock down was confirmed by using a human specific HGF ELISA. WT, KD and NS ctrl MSC supernatants were examined for expression of the HGF protein. Protein expression was calculated utilising a standard curve (A). All cells were characterised by looking at surface marker expression of a variety of cell surface markers against corresponding isotypes by flow cytometry (B).
